# Breast Cancer Screening in Semi-Rural Malaysia: Utilisation and Barriers

**DOI:** 10.3390/ijerph182312293

**Published:** 2021-11-23

**Authors:** Devi Mohan, Tin Tin Su, Michael Donnelly, Wilfred Mok Kok Hoe, Désirée Schliemann, Min Min Tan, Daniel Reidpath, Nur Aishah Taib, Pascale Allotey

**Affiliations:** 1 Global Public Health, Jeffrey Cheah School of Medicine and Health Sciences, Monash University Malaysia, Bandar Sunway 47500, Malaysia; TinTin.Su@monash.edu (T.T.S.); wilfredmok89@gmail.com (W.M.K.H.); daniel.reidpath@icddrb.org (D.R.); pascale.allotey@unu.edu (P.A.); 2 South East Asia Community Observatory (SEACO), Jeffrey Cheah School of Medicine and Health Sciences, Monash University Malaysia, Segamat 85000, Malaysia; tan.minmin@monash.edu; 3 Centre of Public Health, Queen’s University Belfast, Belfast BT12 6BA, UK; michael.donnelly@qub.ac.uk (M.D.); D.Schliemann@qub.ac.uk (D.S.); 4 International Centre for Diarrheal Disease Research, Bangladesh (icddr,b), Dhaka 1212, Bangladesh; 5 Department of Surgery, Faculty of Medicine, Universiti Malaya Cancer Research Institute, University of Malaya, Kuala Lumpur 50603, Malaysia; 6 International Institute for Global Health, United Nations University, Kuala Lumpur 56000, Malaysia

**Keywords:** breast cancer screening, mammogram, clinical breast examination, barriers

## Abstract

Breast cancer (BC) is the commonest cancer in Malaysia. Delayed diagnosis is a significant cause of BC mortality in the country. Early diagnosis and screening are vital strategies in mortality reduction. This study assessed the level of utilisation and barriers for breast self-examination (BSE), clinical breast examination (CBE) and mammogram in a semi-rural population in Malaysia and compared these across the different ethnic groups. This cross-sectional study was conducted among women aged 40 years and above, embedded within a health and demographic surveillance site (HDSS) in Segamat, Malaysia. Trained data collectors collected data on screening and barriers during home visits. Study participants (*n* = 250) were aged 59.4 ± 10.9 years and represented Malaysia’s three major ethnic groups. Practice of regular BSE, CBE uptake (ever) and mammogram (ever) was 23.2%, 36% and 22.4%, respectively. Regular BSE practice was highest in the Malay ethnic group and least among the Chinese. Regular CBE was very low in all ethnic groups (<5%). Mammogram uptake was highest among Chinese (34.4%), followed by Indians (30.4%) and Malays (16.6%). After adjusting for other socio-demographic variables, Malay ethnicity was positively associated with regular BSE (adjusted OR = 5.26, 95% CI 2.05, 13.50) and negatively associated with having had a mammogram (adjusted OR = 0.3, 95% CI 0.15, 0.57). Lower education was negatively associated (adjusted OR = 0.36, 95% CI 0.17, 0.74) with mammogram attendance (ever). Emotional and financial barriers were the most reported types of barriers, specifically, fear of diagnosis (74.8%), cost of diagnosis (69.6%) and fear of losing a breast (66.4%). Malay women more commonly reported most barriers compared to other ethnic groups. Screening uptake was low among semi-rural women in Malaysia. Implementing culturally appropriate interventions that consider ethnic differences is crucial to empowering women to engage in BC screening initiatives in these communities.

## 1. Introduction

Breast cancer (BC) is the commonest cancer and the leading cause of cancer mortality in women globally [1]. In Malaysia, BC is the most frequently diagnosed cancer accounting for nearly 19% of all cancer cases reported to the National Cancer Registry (NCR) [2]. It is the most common cancer among women of all ethnic groups; the highest age-specific incidence is in the age group 45 to 69 years.

BC’s age-standardised incidence and mortality rates in high-income countries are 81 and 12.9 per 100,000 population, respectively. Malaysia has a much higher mortality rate (20.7/100,000) despite a lower incidence of 44/100,000. The five-year survival rate in Malaysia in 2018 was 66.8%, which was lower than that for developed countries [1]. This difference is primarily due to delayed diagnosis and, hence, poorer survival. In Malaysia, 48% of BC cases are diagnosed at an advanced stage (Stages 3 and 4) [2]. Malaysia is a multi-ethnic country, and there are ethnic variations in the incidence and prognosis of BC. The lifetime risk of being diagnosed with BC is highest among Chinese, followed by Indians and Malay [2]. Even though Malay women have the lowest incidence of BC, they have the poorest relative five-year survival (57.9%) compared to Chinese (76.5%) and Indians (70.5%) [3]. Women from rural areas also present at later stages. Sixty per cent of rural women present with advanced BC compared to a national average of 48% [4,5].

BC screening (mammography and clinical breast examination (CBE)) and early diagnosis are proven strategies for BC control and mortality reduction [6]. BC can be clinically detected in early stages, and thus CBE plays a role in screening and improving survival rates in women aged 50 years and older [7]. Although mammography is the gold standard BC screening technique in the developed world, CBE is cost-effective for detecting and down-staging BC in low resource settings [7,8]. Breast self-examination (BSE) is ineffective in reducing BC mortality in a population with early clinical disease. However, it is taught to women as a method to increase breast health awareness [6].

BC screening in Malaysia is opportunistic; the Malaysian Clinical Practice Guidelines recommend that biennial mammograms should be performed among women aged 50 to 74 years; women aged 40–49 years are not offered mammograms routinely. BSE is only recommended as a breast awareness measure. The government provides subsidised mammograms through the Ministry of Women and Family Development (LPPKN) and state government programmes (e.g., Mammosel programme https://skw.yawas.my/web/ (accessed on 22 November 2021)). The level of BC screening utilisation (both CBE and mammogram) in Malaysia is low [9], and is influenced by educational level, socioeconomic status, cultural perception and beliefs of women and the community [10].

There are also disparities in breast health-seeking and BC screening across women from urban and rural areas and different ethnicities in Malaysia. For instance, the uptake of CBE ranges from 37% in indigenous women to 60% among Chinese [11]. Differences in awareness and health-seeking behavior between women from the three ethnic groups in Malaysia are likely contributing to disparities in early detection and survival.

There is a need to improve the understanding of uptake and barriers to breast health-seeking and screening, which are limited, especially in rural areas. It is essential also to understand ethnic differences in terms of barriers to breast health-seeking to develop equitable programs and services that provide culturally appropriate and affordable screening and diagnostic pathways. This study aimed to identify the practice of BSE and BC screening and assess the barriers to BC detection among women aged 40 years and above from different ethnic backgrounds in a semi-rural setting in Malaysia.

## 2. Materials and Methods

### 2.1. Study Design and Setting

This cross-sectional study was embedded within the South East Asia Community Observatory (SEACO). SEACO is a health and demographic surveillance site (HDSS) located in a semi-rural community in Segamat, Johor, in the southern tip of Peninsular Malaysia. SEACO covers five sub-districts of the Segamat district, namely, Bekok, Chaah, Gemereh, Jabi and Sungai Segamat. A baseline enumeration was conducted in 2012–2013, covering 44,902 people with an ethnic representation of Malays, Chinese, Indians and Orang Asli (indigenous population) [12]. Individuals are revisited for an update round every year, and every five years, a more detailed health profile is collected on all consenting participants. The current study drew participants from the 2014 health round (*n* = 25,184).

### 2.2. Study Participants and Sample

Women, 40 years and older, who participated in the SEACO health round (2014) were eligible for inclusion in this study. Based on the estimated uptake of any breast screening (70%) [13], an acceptable error margin of 5%, and a power of 90%, the sample size was calculated as 227. Accounting for a 10% non-response rate, the total sample size was rounded off to 250. These women were selected from the database by stratified random sampling based on ethnicity (proportionate to the national ethnic distribution). Women who did not consent and who were acutely ill during the visit were excluded.

### 2.3. Data Collection

The data collection was carried out between June and September 2017. Trained data collectors conducted home visits and recorded participant responses with a handheld electronic device. After obtaining informed consent, the interviewers collected data using a pre-tested questionnaire using the Survey CTO application (https://www.surveycto.com/ (accessed on 20 November 2021)) in the local language of participant’s preference (Malay, Chinese or English). Field supervisors conducted a regular random observation of interviews to ensure high data quality. The following data were collected during the interviews.

#### 2.3.1. Socio-Demographic Variables

The participants were asked about their age, ethnicity, level of education (no schooling or completed primary, secondary or pre-University education), current marital status, employment status and monthly household income in Malaysian Ringgit (RM, 1RM~0.24 USD).

#### 2.3.2. Breast Self-Examination and Cancer Screening Practices

BC-related information was collected, including previous history and family history of BC and awareness and attitude to BC as a disease.

- BSE: Data on awareness, frequency and timing of BSE, the level of confidence and the perceived benefit of BSE were collected. Women who performed BSE at least once in their lifetime were considered ‘having ever done BSE’, while women who performed BSE at least once every month were regarded as ‘having regular BSE’.
- CBE: Information on CBE awareness was assessed by asking, “Have you heard of Clinical Breast Examination?” and utilisation of CBE was captured by asking, “Has a health care provider ever examined your breasts?” The participants were also asked if they had a CBE in the past year and the number of times they had a CBE in the past five years. At least once a year for the past five years was considered as regular CBE. Access to CBE among women who were aware of a facility was assessed as the reported time in minutes it would take the woman to travel one way to the nearest health clinic.
- Mammogram: Information on the awareness of mammograms was assessed by asking, “Have you heard of mammograms?” and utilisation of mammograms was captured by asking, “Have you ever undergone breast imaging or mammogram?” The participants were also asked if they had undergone a mammogram in the past two years. Women who had a mammogram at least once in their life were categorised as ‘having ever had a mammogram’. Those who had a mammogram in the past two years were regarded as ‘having done a recent mammogram’ [13]. Access to mammograms was assessed as the time in minutes it would take for a woman to travel one way to the nearest health clinic with a mammogram facility (among women who were aware of such a facility).

#### 2.3.3. Perceived Benefit and Barriers

The perceived benefit of all screening methods was independently assessed by asking the question, “Do you think BSE/CBE/mammogram helps with early detection and better outcome of breast cancer?” (yes/no). Data was also collected on the awareness and utilisation of BC screening programmes offered by government agencies (subsidised programme by LPPKN) and non-governmental organisations (NGOs). Self-reported barriers were collected from the participants on 15 items related to breast health-seeking and BC screening, on a five-point Likert scale. These variables were dichotomised to yes (strongly agree, agree) and no (not sure, disagree and strongly disagree). The barriers were categorised into five domains, namely emotional (3 items), practical/financial (4 items), health system-related (4 items), sociocultural (2 items) and screening related (2 items) [14].

### 2.4. Ethical Considerations

The study was approved by Monash Human Research Ethics Committee (No. 7844). Data collectors obtained informed written consent from all participants. Women with self-reported BC warning signs were referred to the BC center at the District Hospital.

### 2.5. Data Analysis

Data were analysed using SPSS version 24. Quantitative variables were reported as mean and standard deviation (SD) for normally distributed variables; and median and interquartile range (IQR) for variables with non-normal distribution. Categorical variables were expressed as frequency (*n*) and percentage (%). Associations between categorical variables and comparison of proportions were performed using the Chi-square test. A binary logistic regression was performed for multivariate analysis for socio-demographic factors associated with screening practices. All socio-demographic variables with a *p* value ≤ 0.2 in bivariate analysis were included in the logistic regression model. For bivariate and multivariate analysis, regular BSE, ever performed CBE and ever undergone mammogram were used as outcome variables. All statistical tests were interpreted at a significance level of 5%.

## 3. Results

### 3.1. Socio-Demographic Characteristics

We approached a total of 270 eligible women to obtain a sample size of 250 (response rate of 92.6%). Characteristics of these participants are presented in Table 1. The participants’ mean age (SD) was 59.4 (10.9) years, and 90.4% had at least a primary education. Women from the Malay ethnic group made up 65.2% of the sample. Most of the participants were married (68%) and homemakers (75.2%). Nearly one-third (32%) of the women had a monthly family income below the minimum wage in Malaysia during the year of data collection. (RM 1000~USD 247). Twenty-seven women (10.8%) had a positive family history of BC, and two women were previously diagnosed with BC.

### 3.2. Knowledge and Attitude towards BC and Breast Help-Seeking

Most women were aware that BC is a health problem in the community (95.2%) and can be fatal if left untreated (94%), but only 67.6% believed that the disease was preventable. Husbands (52%) and daughters (26.8%) were the people the women would first talk to if they had any BC symptoms. Ninety-two per cent of women would seek help from a physician if they had any BC symptoms, and 83.2% would contact the physician at least within a few days.

### 3.3. Awareness, Access and Utilisation of Breast Self-Examination BC Screening Practices

- BSE: As shown in Table 2, 72% of women have had BSE at least once, but only 23.2% performed BSE regularly. More than half of the women (56%) reported needing more training to perform BSE. Among women who were aware of BSE (*n* = 195), 86.2% perceived BSE to be beneficial.
- CBE: Among the 250 women, 133 (53.2%) were aware of CBE as a BC screening method, while only 36% had ever undergone a CBE (Table 2), and 4.4% performed CBE regularly. CBEs were conducted by doctors (72.2%) and community health nurses (27.8%). Most of the recent CBEs were completed to screen for BC (80.6%) and the remaining CBEs were related to pregnancy or other breast health issues. Only 46% of women were aware of the nearest health facility that offered CBE. The median travel time (IQR) to reach the facility was reported as 15 (20) min, and more than 25% of the women had a travel time of ≥30 min. Among the women aware of CBE, 126 (94.7%) believed that CBE was beneficial.
- Mammogram: Only 47.6% (*n* = 119) of women had heard of mammograms as a BC screening method, and 22.4% of women received a mammogram at least once in their lifetime (Table 2). Few participants (10.8%) received a mammogram recently. Out of these mammograms, 88.9% were done to screen for BC, and 48.1% had their mammogram in a private facility. Among women who were aware of mammograms, 95.8% reported that they believed mammogram was beneficial. Only 21.2% of the participants knew the nearest health facility that offered a mammogram. These women reported a median (IQR) time of 70 (72.5) minutes to reach the mammogram facility.
- Breast cancer screening programmes: Among all interviewed women, only 15.6% were aware of the existing mammogram subsidy offered by LPPKN and only 8% used this subsidy. Only 11.2% of women utilised any form of BC screening programme offered by NGOs.

### 3.4. Ethnic Difference in Screening Practices

**Figure 1:**
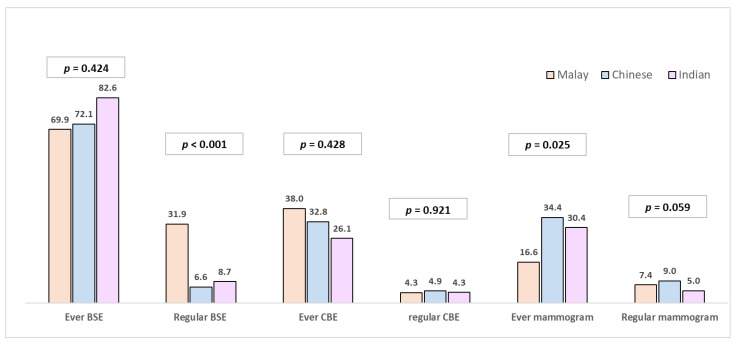
Breast cancer screening practices among women from different ethnic groups (percentage), *n*: Malay = 163, Chinese = 61, Indian = 23; *p* values reported for chi-square test comparing the three ethnic groups.

Figure 1 shows the difference in screening practices between the three major ethnic groups—Indian women most commonly reported to have ever carried out BSE. However, Malay women most commonly reported performing BSE regularly, followed by Indian and Chinese women (31.9%, 8.7% and 6.6%, respectively, *p* < 0.001). More Malay ethnic women also reported to have ever had a CBE (38%) compared to Chinese (32.8%) and Indian (26.1%) women; however, the difference was not significant. Less than 5% of women from all ethnic groups attended CBE regularly. Mammogram utilisation was highest among Chinese women, followed by Indian and Malay (34.4%, 30.4% and 16.6%, respectively, *p* = 0.025).

### 3.5. Socio-Demographic Factors Associated with Screening Practice

Results of the bivariate and multivariate analyses for socio-demographic factors associated with BSE (regularly), CBE (ever) and mammogram (ever) practice are presented in Table 3. After adjusting for confounders, Malay ethnicity (adjusted OR = 5.26, 95% CI 2.05, 13.50) and a household income of ≥RM 1000 (adjusted OR = 2.91, 95% CI 1.31, 6.44) were positively associated with regular BSE. Women who were married had higher odds of ever having had a CBE (adjusted OR = 2.28, 95% CI 1.20, 4.31) than unmarried or divorced women. Women with primary or lower education were 64% less likely to have had a mammogram (adjusted OR = 0.36, 95% CI 0.17, 0.74) than those with at least secondary education. Being married was positively associated with having had a mammogram (adjusted OR = 2.33, 95% CI 1.08, 5.03), and Malay ethnicity was negatively associated with having had a mammogram (adjusted OR = 0.30, 95% CI 0.15, 0.57).

### 3.6. Barriers to BC Screening and Health-Seeking

**Figure 2:**
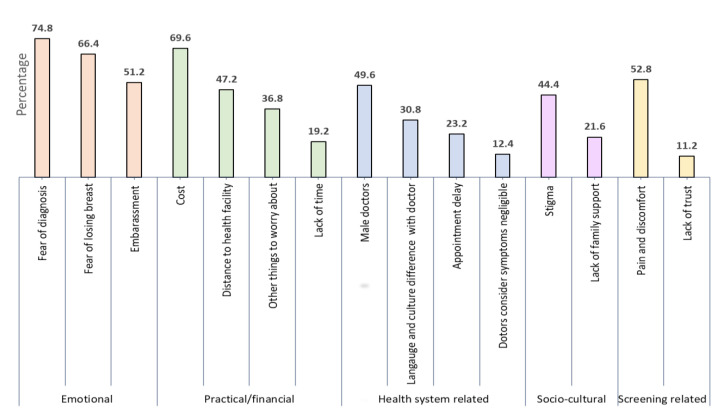
Perceived barriers reported by women expressed in percentage (*n* = 250).

Self-reported barriers to screening and breast health are shown in Figure 2. Fear of diagnosis (74.8%), cost (69.6%) and fear of losing a breast (66.4%) were the commonest reported barriers. Emotional barriers were the most common, with all emotional barriers reported by more than half of the participants. Pain and discomfort associated with screening (52.8%), embarrassment (51.2%), having to approach a male doctor (49.6%), distance to the health facility (47.2%), and stigma (44.4%) were also commonly reported barriers.

Table 4 shows the differences in perceived barriers between ethnic groups. ‘Fear of diagnosis’ significantly differed between ethnic groups and was most reported by Malay women, followed by Chinese and Indian women (79.8%, 67.2% and 56.5%, respectively, *p* = 0.018). Significantly more Malay women reported the barriers (in descending order): ‘cost’ (76.1%), ‘pain and discomfort’ (62.6%) ‘uncomfortable with a male doctor’ (58.9%), ‘distance to healthcare facility’ (54%), ‘lack of family support’ (28.8%), and ‘delay in getting appointment’ (28.8%), ‘lack of time’ (27%), and ‘doctors might consider symptoms as negligible’ (17.2%) compared to Indian and Chinese women.

## 4. Discussion

Our study shows an overall low utilisation of opportunistic mammographic and CBE screening. Furthermore, emotional barriers (fear of diagnosis and losing a breast, embarrassment), costs, fear of pain and discomfort, feeling uncomfortable with male doctors and distance to healthcare facilities were important barriers.

This study is the first to include a representative sample of multi-ethnic semi-rural women with a relatively low level of education from a lower socioeconomic status. The proportion of women in the study who were aware and had practiced BSE (ever) were high, however, the practice of regular BSE was low. Less than one in four women performed monthly BSE compared to around 40% reported by studies among predominantly urban women with higher education [15]. Although regular BSE was low across all ethnic groups, the Malay ethnic women reported the highest BSE rates. Four times as many Malay women conducted regular BSE as Indian women or Chinese women. However, Malay ethnicity is associated with late-stage presentation and lower survival rates [16]. Thus, notably, there is an opportunity to empower women to seek help early after the detecting breast changes. Most women in our study thought BSE was beneficial, but more than half felt they needed more training. These findings suggest that women in semi-rural areas have a positive attitude toward BSE but require the confidence to perform breast examinations regularly and overcome other barriers like cost, worry about pain and discomfort, and embarrassment to come forward at the earliest signs of breast changes.

In addition, our study also found that higher income was positively associated with regular BSE. It was also observed in the National Health and Morbidity Survey 2019, that Malay women and women from the highest income group had higher rates of BSE in the past year [9]. These findings suggest that some Malay women understand that there is a health threat. However, this perceived threat did not appear to translate to action or the use of standard diagnostic services. Malay women notably had lower utilisation rates of mammograms compared to other ethnic groups.

Regular CBE for BC screening purposes in our study was low in all ethnic groups. Malay women were also more commonly reported to have had CBE than Chinese and Indians, but this ethnic difference did not exist for regularly having CBE. The higher parity among Malays may suggest that some of the CBEs may have been conducted during routine antenatal check-ups. In 2010, the Malaysian National Technical Committee on preventing BC recommended CBE as the primary modality for BC screening with a target to screen 50% of high-risk women and 90% of all women attending the Ministry of Health (MOH) government community clinics [17]. Mammogram screening was only recommended for targeted screening of high-risk women. The MOH Family Health Division Report in 2019 reported that 29.2% of women aged 20 years and above had received CBE. This trend has been increasing over the years. However, 73.3% of the CBEs were in the age group 20–39 years, while only 2.7% were among women aged 40 years and older, which indicates that we are missing a significant proportion of older women at risk of BC [18].

Emotional barriers, cost and pain and discomfort of screening were the commonest reported barriers in our study. A qualitative study conducted by Lim et al. in Malaysia and Singapore found that women delayed seeking help for breast-related symptoms due to fear of pain, fear of diagnosis, fear of treatment and financial challenges [14]. Emotional barriers, poor literacy and lack of financial and social capital were also significant barriers to seeking help for cancer in an urban population [19]. However, according to another study conducted in a tertiary care facility in an urban area, cost was not a barrier [10]. The fear of cost in our study population could be due to the higher reliance on private health facilities or projected costs of diagnosis and treatments. Malay women expressed more fear of diagnosis and fear of pain and discomfort compared to their Chinese and Indian counterparts. Malay women also reported the highest level of lack of support from family in screening and breast health-seeking.

There were significant ethnic differences in the health system-related barriers. Compared to other ethnic groups, twice the proportion of Malay women reported the discomfort of seeking care from a male doctor for breast screening. This finding is in line with Mahmud et al.’s systematic review results, which suggested that the presence of male staff was a significant barrier for mammography. All these barriers could explain the lower rates of regular CBE and mammogram among Malay women resulting in late presentation and poorer survival.

The participants in our study had low levels of awareness and experience in utilising existing government and local NGO BC screening programmes. This might be due to a lack of campaigning or a higher travel burden to access these services [20]. It is positive to note that only a small proportion of women aware of BC screening lacked trust in screening practices. The perceived benefit of BSE (86.2%), CBE (94.7%) and mammogram (95.8%) among those who were aware of it was very high. These findings highlight that the lack of awareness of screening methods, access and subsidised programmes contribute to low screening uptake, rather than women’s perception or acceptance of these modalities.

In our study, less than half of the women knew that nearby health facilities (in our research, the average travel time was only 15 min) could perform CBE. Despite the accessibility of CBE services, the awareness about the screening methods and their utilisation was poor. Future BC screening interventions should prioritise increasing awareness about screening and health-seeking in primary care facilities for CBE in semi-rural communities in Malaysia, a country with one of the world’s best primary health care system [21]. Providing population-wide CBE would require routine biennial visits with a robust post-screening diagnostic pipeline that is cost-effective, given the wide and ready access to the Ministry of Health and Ministry of Women Family and Community Development community clinics.

Mammograms were the least common cancer screening method in our study; less than 25% had ever had mammograms, which was comparable to data from NHMS for rural areas in the country [9]. Furthermore, the prevalence of recent mammograms was very low (10.8%) compared to the national average of 21% [9]. Our study found that women had poor knowledge and physical access (median travel time of 70 min) to facilities with a mammogram. Previous studies in Malaysia showed that women from rural areas have a higher travel burden to mammogram facilities than in urban areas [20]. According to a study conducted in the US, women are unlikely to attend mammogram screening if they have to travel more than 20 miles [22]. It is also noteworthy that half of the women performed mammograms in a private facility, much higher than the national utilisation level of 37%. (9) Improving awareness and access to mammograms in public health care settings may increase coverage in rural areas. The uptake was highest among Chinese and lowest among Malay ethnic women. A similar utilisation pattern was also observed in other studies conducted in Malaysia [9,23] and could be due to the differences in socioeconomic and sociocultural beliefs and health-seeking behavior. Primary or lower levels of education were negatively associated with mammogram uptake among our study participants, which is consistent with other studies conducted in similar settings [24,25].

Our study clearly shows the low BC screening uptake and its most important barriers in a semi-rural community in Malaysia. Studies in other parts of the world have identified that culturally sensitive interventions should be developed to overcome barriers and increase BC screening among women from diverse cultural backgrounds. Hence, we should develop targeted interventions to accommodate the ethnic differences and address access and financial support issues to increase BC screening uptake in the rural regions.

## 5. Strengths and Limitations

While many studies in Malaysia have studied the uptake of BC screening practices, most of them focused on the urban population and specific ethnic groups. Ours is the first study to evaluate the ethnic differences in screening practices and barriers among a representative semi-rural population in the southern state of Johor in Malaysia. The study was embedded in the ongoing longitudinal study of the SEACO HDSS. It also offers the ease of follow-up and future interventions due to the longitudinal nature of the HDSS.

Our study, however, has a few limitations. Firstly, the study was conducted in a semi-rural population and cannot be generalised to the entire Malaysian population. This is not unexpected, as the purpose of HDSS is not for generalisation but to understand a health issue, its trend, and patterns in a specific geographical area [12]. Secondly, our sample size was primarily based on studying the utilisation of screening practices. As such, the study may not have enough power to detect differences across groups. However, findings on ethnic differences observed from other studies are similar to the pattern observed in our study [9,25]. Thirdly, the data was collected in 2017 and, hence, not recent. Fourthly, our study was conducted among women aged 40 years and above who have the highest incidence of BC in Malaysia. It does not reflect the BC screening pattern among younger women (less than 40 years)

## 6. Conclusions

BC self-reported screening practices were low in this semi-rural community. Despite adequate knowledge and attitudes to BC screening modalities, emotional, financial, and physical factors were significant barriers to BC early detection in this population. We found substantial ethnic differences in the practices and barriers of BC screening practices. Thus, culturally adapted community-based intervention that addresses health literacy, emotional, cost and access barriers need to be developed urgently.

## Figures and Tables

**Table 1:** Characteristics of study participants (*n* = 250).

Characteristics	Frequency (%)
**Age** (in years), mean (SD) Below 60 60 and above	59.4 (10.9) 128 (51.2) 122 (48.8)
**Education**	
No schooling	24 (9.6)
Primary	121 (48.4)
Secondary	98 (39.2)
Pre-university/university	7 (2.8)
**Ethnicity**	
Malay	163 (65.2)
Chinese	61 (24.4)
Indian	23 (9.2)
Others	3 (1.2)
**Marital status**	
Currently married	170 (68)
Widow Separated/divorced Unmarried	80 (32) 6 (2.4) 9 (3.6)
**Employment status**	
Currently working Homemaker	36 (19.6) 188 (75.2)
Unemployed Retired	13 (5.2) 13 (5.2)
**Household income** (monthly, RM) Median (IQR) (*n* = 221)	1200 (875)
At least minimum wage ^#^ (≥RM 1000) Below minimum wage (<RM 1000)	141 (56.4) 80 (32)
**Family history of breast cancer**	27 (10.8)
**A previous breast cancer diagnosis**	2 (0.8)

# Minimum wage in the year 2017.

**Table 2:** Awareness, utilisation, and access to breast cancer screening modalities among the study participants.

Awareness, Utilisation and Access Measures	Breast Self-Examination	Clinical Breast Examination	Mammogram
	Frequency (%)	Frequency (%)	Frequency (%)
Have heard about this screening	195 (78)	133 (53.2)	119 (47.6)
Have ever had this screening	180 (72)	90 (36)	56 (22.4)
Have undergone this screening regularly	58 (23.2)	11 (4.4)	27 (10.8) ^a^
Perceived benefit of this screening method ^b^	168 (86.2)	126 (94.7)	114 (95.8)
One-way trip length to the health facility ^c^		(*n* = 115)	(*n* = 53)
<30 min	NA	86 (74.8)	14 (26.4)
30 min to 60 min		24 (20.9)	20 (37.7)
>60 min		5 (4.3)	19 (35.8)

^a^ Having had a recent mammogram (in the past two years) was considered regular. ^b^ Analysed as a proportion of women who were aware of the screening method. ^c^ Assessed only among women who were aware of the nearest health facility offering the screening service.

**Table 3:** Socio-demographic factors associated with breast cancer screening practices.

Socio-Demographic Variables	Regular BSE				CBE				Mammogram			
	Yes (Freq,%)	No (Freq,%)	*p* Value ^a^	*p* value ^b^ Adjusted OR (95% CI)	Ever (Freq, %)	Never (Freq, %)	*p* value ^a^	*p* value ^c^ Adjusted OR (95% CI)	Ever (Freq, %)	Never (Freq, %)	*p* value ^a^	*p* value ^d^ Adjusted OR (95% CI)
**Age**			0.013	0.342			0.002	0.148				
Below 60	38 (65.5)	90 (46.9)			58 (64.4)	70 (43.8)			33 (58.9)	95 (49)	0.189	0.560
60+ (Ref)	20 (34.5)	102 (53.1)			32 (35.6)	90 (56.3)			23 (41.1)	99 (51)		
**Education**			<0.001	0.137			0.003	0.114			0.009	0.005 0.36 (0.17, 0.74)
Primary and below	21 (36.2)	124 (64.6)			41 (45.6)	104 (65)			24 (42.9)	121 (62.4)		
Secondary and above (Ref)	37 (63.8)	68 (35.4)			49 (54.4)	56 (35)			32 (57.1)	73 (37.6)		
**Ethnicity**			<0.001	0.001 5.26 (2.05, 13.50)			0.358	NA			0.002	<0.001 0.30 (0.15–0.57)
Malay	52 (89.7)	111 (57.8)			62 (68.9)	101 (63.1)			27 (48.2)	136 (70.1)		
Non-Malay (Ref)	6 (10.3)	81 (42.2)			28 (31.1)	59 (36.9)			29 (51.8)	58 (29.9)		
**Marital status**			0.143	0.571			0.001	0.012 2.28 (1.20, 4.31)			0.024	0.031 2.33 (1.08, 5.03)
Currently married	44 (75.9)	126 (65.6)			73 (81.1)	97 (60.6)			45 (80.4)	125 (64.4)		
Unmarried/widowed (Ref)	14 (24.1)	66 (34.4)			17 (18.9)	63 (39.4)			11 (19.6)	69 (35.6)		
**Household income**			0.001	0.008 2.91 (1.31, 6.44)			0.369	NA			0.627	NA
At least minimum wage	47 (81)	94 (57.7)			58 (67.4)	83 (61.5)			34 (66.7)	107 (62.9)		
Below minimum wage	11 (19)	69 (42.3)			28 (32.6)	52 (38.5)			17 (33.3)	63 (37.1)		

BSE: breast self-examination; CBE: clinical breast examination; OR: odd’s ratio, CI: confidence interval; Freq (%): frequency and percentage. ^a^ Chi square test; ^b^ logistic regression model with independent variables age, education, ethnicity, marital status, household income; ^c^ logistic regression model with independent variables age, education and marital status; ^d^ logistic regression model with independent variables age, education, ethnicity, marital status; NA: not included in the logistic regression model.

**Table 4:** Ethnic differences in the perceived barriers for breast cancer screening and breast help seeking.

Type of Barriers	Perceived Barriers	Total (*n* = 250) Frequency (Percentage) *	Malay (*n* = 163) Frequency (Percentage) *	Chinese (*n* = 61) Frequency (Percentage) *	Indians (*n* = 23) Frequency (Percentage) *	*p* Value ^#^
**Emotional**	Fear of diagnosis	187 (74.8)	130 (79.8)	41 (67.2)	13 (56.5)	**0.018**
	Fear of losing breast	166 (66.4)	112 (68.7)	38 (62.3)	13 (56.5)	0.401
	Embarrassment	128 (51.2)	91 (55.8)	24 (39.3)	11 (47.8)	0.085
**Socio-cultural**	Stigma on breast health seeking	111 (44.4)	76 (46.6)	26 (42.6)	7 (30.4)	0.330
	Lack of family support	54 (21.6)	47 (28.8)	5 (8.2)	2 (8.7)	**0.001**
**Health system related**	Uncomfortable with male doctors	124 (49.6)	96 (58.9)	19 (31.1)	7 (30.4)	**<0.001**
	Doctors might consider symptoms as negligible	31 (12.4)	28 (17.2)	3 (4.9)	0	**0.008**
	Delay in getting appointment	58 (23.2)	47 (28.8)	9 (14.8)	2 (8.7)	**0.018**
	Doctors do not understand the language and culture	77 (30.8)	47 (28.8)	24 (39.3)	5 (21.7)	0.195
**Practical/** **financial**	Other things to worry about	92 (36.8)	62 (38.0)	23 (37.7)	6 (26.1)	0.532
	Lack of time	48 (19.2)	44 (27.0)	2 (3.3)	1 (4.3)	**<0.001**
	Cost	174 (69.6)	124 (76.1)	34 (55.7)	13 (56.5)	**0.005**
	Distance to health care facility	118 (47.2)	88 (54.0)	20 (32.8)	8 (34.8)	**0.009**
**Screening-related**	Lack of trust in screening	28 (11.2)	24 (14.7)	3 (4.9)	1 (4.3)	0.065
	Pain and discomfort	132 (52.8)	102 (62.6)	21 (34.4)	7 (30.4)	**<0.001**

# Chi-square test, * percentage of women who reported each barrier.

## Data Availability

The data presented in this study are available on request from the corresponding author. The data are not publicly available due to ethical concerns.
